# Molecular features of a human rhabdomyosarcoma cell line with spontaneous metastatic progression

**DOI:** 10.1054/bjoc.1999.1069

**Published:** 2000-02-21

**Authors:** F A Scholl, D R Betts, F K Niggli, B W Schäfer

**Affiliations:** Divisions of Clinical Chemistry & Biochemistry and Oncology, Department of Paediatrics, University of Zurich, Zurich, CH-8032, Switzerland

**Keywords:** rhabdomyosarcoma, myogenic transcription factors, PAX, metastasis, cytogenetic alteration

## Abstract

A novel human cell line was established from a primary botryoid rhabdomyosarcoma. Reverse transcription polymerase chain reaction investigations of this cell line, called RUCH-2, demonstrated expression of the regulatory factors PAX3, Myf3 and Myf5. After 3.5 months in culture, cells underwent a crisis after which Myf3 and Myf5 could no longer be detected, whereas PAX3 expression remained constant over the entire period. Karyotype analysis revealed breakpoints in regions similar to previously described alterations in primary rhabdomyosarcoma tumour samples. Interestingly, cells progressed to a metastatic phenotype, as observed by enhanced invasiveness in vitro and tumour growth in nude mice in vivo. On the molecular level, microarray analysis before and after progression identified extensive changes in the composition of the extracellular matrix. As expected, down-regulation of tissue inhibitors of metalloproteinases and up-regulation of matrix metalloproteinases were observed. Extensive down-regulation of several death receptors of the tumour necrosis factor family suggests that these cells might have an altered response to appropriate apoptotic stimuli. The RUCH-2 cell line represents a cellular model to study multistep tumorigenesis in human rhabdomyosarcoma, allowing molecular comparison of tumorigenic versus metastatic cancer cells. © 2000 Cancer Research Campaign


*British Journal of Cancer* (2000) **82**(6), 1239–1245
© 2000 Cancer Research Campaign
Article no. bjoc.1999.1069, available online at http://www.idealibrary.com on IDEAL®

### Molecular features of a human rhabdomyosarcoma cell line with spontaneous metastatic progression

**FA Scholl[1], DR Betts[2], FK Niggli[2] and BW Schäfer[1]**

Divisions of [1]Clinical Chemistry & Biochemistry and [2]Oncology, Department of Paediatrics, University of Zurich, CH-8032 Zurich, Switzerland

**Summary** A novel human cell line was established from a primary botryoid rhabdomyosarcoma. Reverse transcription polymerase chain reaction investigations of this cell line, called RUCH-2, demonstrated expression of the regulatory factors PAX3, Myf3 and Myf5. After 3.5 months in culture, cells underwent a crisis after which Myf3 and Myf5 could no longer be detected, whereas PAX3 expression remained constant over the entire period. Karyotype analysis revealed breakpoints in regions similar to previously described alterations in primary rhabdomyosarcoma tumour samples. Interestingly, cells progressed to a metastatic phenotype, as observed by enhanced invasiveness in vitro and tumour growth in nude mice in vivo. On the molecular level, microarray analysis before and after progression identified extensive changes in the composition of the extracellular matrix. As expected, down-regulation of tissue inhibitors of metalloproteinases and up-regulation of matrix metalloproteinases were observed. Extensive down-regulation of several death receptors of the tumour necrosis factor family suggests that these cells might have an altered response to appropriate apoptotic stimuli. The RUCH-2 cell line represents a cellular model to study multistep tumorigenesis in human rhabdomyosarcoma, allowing molecular comparison of tumorigenic versus metastatic cancer cells. © 2000 Cancer Research Campaign

**Keywords:** rhabdomyosarcoma; myogenic transcription factors; PAX; metastasis; cytogenetic alteration

Rhabdomyosarcoma (RMS), a solid tumour of skeletal muscle origin, is the most common soft tissue tumour in children, accounting for approximately 5–8% of all paediatric neoplasias (Pappo, 1995). Histologically, it can be divided into four categories: embryonal, botryoid, alveolar, or pleomorphic rhabdomyosarcoma (Newton et al, 1995). The embryonal type (eRMS) is more prevalent and accounts for 50–60% of all cases. It has a more favourable prognosis affecting predominantly children under 15 years of age in the head-and-neck region. It is characterized by frequent loss of heterozygosity on chromosome 11p15.5 (Scrable et al, 1987), expression of myogenic regulatory factors (Tonin et al, 1991) and deregulated expression of PAX3 and/or PAX7 (Schäfer et al, 1994). Botryoid RMS, a subgroup of the embryonal type with usually the best prognosis, is almost exclusively found in young children. Its frequency is about 4–8% of all RMS. The majority of this RMS type is found in mucosa-lined hollow organs, such as the nasal cavity, nasopharynx, bile duct, urinary bladder and vagina. Alveolar RMS (aRMS), an aggressive tumour occurring in children and young adults, is the second most common type of RMS (18–20%) and is characterized by one of the following specific translocations: t(1;13)(p36;q14) PAX7/FKHR or t(2;13)(q35;q14) PAX3/FKHR (Barr et al, 1993, 1998; Galili et al, 1993; Shapiro et al, 1993; Davis et al, 1994).

Unlike other tissue types, muscle development can serve as paradigm for understanding the molecular basis of cell lineage establishment and differentiation. The current picture of myogenesis is based on the isolation and characterization of mainly two families of transcriptional regulators, the muscle regulatory factors (MRF, including Myf3 (MyoD), Myf4 (myogenin), Myf5 and Myf6 (MRF4)) and the myocyte enhancer factor 2 (MEF2) family (Ludolph and Konieczny, 1995). Since differentiation and proliferation are mutually exclusive events, muscle cells can also serve as a model to investigate cell cycle control. In contrast to normal myoblasts, RMS cells are blocked in the differentiation process and have lost growth control.

*Received 24 March 1999*
*Revised 23 September 1999*
*Accepted 2 November 1999*

*Correspondence to:* BW Schäfer

To facilitate the study of multistage carcinogenesis it is necessary to establish cell cultures that can be used as tumour models in vitro. Although few RMS cell lines are available, each line has its specific advantages and disadvantages. The most common used culture model is the RD cell line that dates from 1969. Like this cell line, many of the RMS cell lines were derived from relapse tumours or metastasis, which might not fully reflect the properties of the original RMS tumour and does not allow to study tumour progression in vitro. One additional problem is an uncertainty regarding the origin of some of the lines available. This is especially true when cells were established from eRMS, have no specific translocations, and lost in some cases expression of the characteristic myogenic regulators like Myf3 (MyoD). Then the generally accepted interpretation would suggest that the original tumour was misdiagnosed and the cells represent a different tumour, e.g. Ewing's sarcoma or primitive neuroectodermal tumour (PNET).

To obtain additional insight into RMS tumour development, we started an initiative to isolate and establish additional well characterized cell lines of RMS origin. Our interest is to find and investigate additional factors which might influence the balance between growth and differentiation in RMS cells, allowing the molecular characterization of the mechanisms implicated in multistage development of this neoplasm. In this work, we describe the isolation of a new human cell line derived from a primary botryoid RMS, and its evolution during time in culture.

### MATERIALS AND METHODS

### Clinical history

The patient was a 15.5-month old white girl. The tumour was diagnosed unequivocally as a botryoid rhabdomyosarcoma in the vagina, belonging to group III of the Intergroup RMS Study Surgical-Pathological Grouping Classification. The immunohistochemistry showed strong staining with antibodies against vimentin, desmin, α-muscle actin and some weak myoglobin. A tumour biopsy was taken before any treatment and used to establish the RUCH-2 cell line. The patient was subsequently treated with standard chemotherapy and is still in remission.

### Mice

Nude mice were maintained under spf (specific pathogens-free) conditions in the animal facility of the University of Zurich (Central Biological Laboratory). Experiments were conducted according to the Swiss ordinance for animal experiments.

### Establishment of the RUCH-2 cell line

Primary tumour fragments from the biopsy were injected subcutaneously (s.c.) into 4- to 6-weeks old female ICR *nu/nu* mice. The xenograft was removed after two passages and minced under aseptic conditions. The tissue pieces were seeded into a 100-mm culture dish containing Dulbecco's modified Eagle medium (DMEM) supplemented with 10% fetal bovine serum (FBS) and 100 units $ml^{-1}$ penicillin 100 μg $ml^{-1}$ streptomycin, and maintained in an incubator at 37°C and 10% carbon dioxide ($CO_2$). The culture medium was changed three times a week. After reaching confluence, tumour cells were passaged 1–3 times a week using trypsin solution. RUCH-2 cells were routinely monitored for mycoplasma and murine cell contamination by fluorescent staining with Hoechst 33258, and found to be free of both.

### Cell lines

Human cell lines were cultured in DMEM supplemented with 10% FBS and 100 U $ml^{-1}$ penicillin and 100 μg $ml^{-1}$ streptomycin in 10% $CO_2$ and 37°C. The embryonal RMS cell line RD was obtained from ATCC (Rockville, MD, USA) (McAllister et al, 1969). The embryonal RMS Rh1 and the alveolar RMS Rh30 cell lines were a generous gift from Dr PJ Houghton (St Jude Children's Research Hospital, Memphis, TN, USA). The alveolar RMS cell line RC2 was kindly provided by Dr C De Giovanni (Istituto di Cancerologia, Bologna, Italy) (Nanni et al, 1986).

### Chromosomal studies

Metaphase preparations were obtained according to standard cytogenetic methods or for the tumour biopsy as previously described (Betts et al, 1997). Along with the original tumour, the RUCH-2 cell line was karyotyped at passages 3, 20, 32, 46, 62, 84 and 104. Cytogenetic analysis and interpretation were made according to ISCN (1995).

### Tumorigenicity of cell lines in vivo

To evaluate the tumorigenic capacity of the RUCH-2 cell line, cells at passage 30 were injected s.c. into the dorsal region of three females C57B1/6 *nu/nu* mice (5–7 weeks old). Each mouse received 100-μl cell suspension containing $5 \times 10^6$ viable cells as determined by trypan blue exclusion. As control an additional mouse was injected with the same amount of FUCH-1 cells (normal human fibroblast cell line of the same patient at passage 6) and two further mice were injected with the same amount of RD cells. The experiment was repeated at passage 67 of the RUCH-2 cell line, whereby ICR *nu/nu* mice (6–8 weeks old) were used. Control FUCH-1 and RD cells grew similarly in both mouse strains.

### Invasion assay

The Biocoat® Matrigel® Invasion Chamber was used to perform invasion assays (Becton Dickinson Labware, Lot 906658). The chambers, placed in a 24-well plate, were rehydrated with 250 μl DMEM for 2 h at room temperature. Single-cell suspensions of different passages of the RUCH-2 cell line were obtained with 5 mM EDTA/PBS (phosphate-buffered saline). The cells were resuspended in 0.1% bovine serum albumin (BSA)/DMEM to a final volume of $1 \times 10^5$ cells $ml^{-1}$. Then, 750 μl 5% fetal bovine serum (FBS)/DMEM (antibiotica-free) was added to the wells as a chemoattractant and 500 μl of cell suspension was added to each chamber. After 22 h non-invasive cells were removed from the upper surface of the membrane with a cotton swab. The chambers were then fixed with ice-cold methanol for 10 min and stained with crystal violet. The membranes were removed, placed on coverslips and invasive cells were counted under the microscope.

### RT-PCR analyses

Reverse transcription polymerase chain reaction (RT-PCR) was performed using the Access RT-PCR System Kit (Promega) in a 25 μl reaction volume with 100 ng total RNA. As positive and negative controls, RNA from the established rhabdomyosarcoma cell lines RD, Rh1, Rh30, or RC2 were used. Total RNA was extracted using guanidinium-isothiocyanate lysis followed by centrifugation through a 5.7 M caesium chloride (CsCl) cushion. All reactions started with reverse transcription at 48°C for 45 min, followed by a 2-min initial denaturation step at 94°C, and 40 cycles at 94°C for 30 s, 60 or 68°C for 1 min, 68°C for 1 min, with a final extension at 68°C for 7 min on a PCR system (Perkin-Elmer Cetus). The 68°C annealing temperature was used only for the Myf-3 amplification. The FKHR, PAX3 and PAX7 products were generated in 0.5 mM magnesium sulphate ($MgSO_4$); the PAX3PAX7/FKHR, Myf-3, and Myf-5 in 1.0 mM $MgSO_4$. The following primers were used to amplify a 324-bp long fragment of FKHR [*FKHR-F5* 5′-GCAGATCTACGAGTGGATGG-3′ and *FKHR-F3* 5′-AACTGTGATCCAGGGCTGTC-3′] (Galili et al, 1993), a 349-bp fragment of PAX3 [*PAX3-5′* 5′-GCACTGTA-CACCAAAGCACG-3′ and *PAX3-REV* 5′-TAGGTGGGTG-GACAGTAGGA-3′], a 414-bp fragment of PAX7 [*PAX7-3* 5′-GGCGTAAGCAGGCAGGAG-3′ and *PAX7-4* 5′-GCAGCGG-GGAGATGGAGA-3′], a 868-bp resp. 862-bp fragment for the translocations PAX3/FKHR resp. PAX7/FKHR [*PAX3PAX7* 5′-CCAAACACAGCATCGACG-3′ (this primer anneals to both PAX3 and PAX7) (Davis et al, 1994) and *FKHR-F3*], a 159-bp fragment of Myf-3 [*MYF3-FOR* 5′-CTGTGGGCCTGCAAG-GCGTGCAAG-3′ and *MYF3-REV* 5′-CACCTTGGGCAACCGC-TGGTTTGG-3′] (Anand et al, 1994), and finally a 612-bp fragment of Myf-5 [*MYF5-FOR1* 5′-AGCCTGCAAGAGGAAGTCC-3′ and

© 2000 Cancer Research Campaign

*MYF5-REV* 5′-AGCCTTCTTCGTCCTGTGTA-3′]. One-third of the RT-PCR products were separated by agarose gel electrophoresis.

### Hybridization of arrays

Atlas™ Human Cancer cDNA Expression Arrays were purchased from Clontech (Lot 8090622). mRNAs (RUCH-2 at passage 11 and passage 63) were obtained by isolation of total RNA (CsCl-centrifugation), followed by treatment with RQ1 RNAase-free DNase I (Promega). Thereafter poly-$A^+$ RNA was isolated with a mRNA isolation kit (Boehringer Mannheim), finishing with a phenol:chloroform:isoamylalcohol (25:24:1) extraction and ethanol precipitation. $^{32}$P-labelled cDNA probes were prepared from 1 μg of mRNA. Both probes, with a final concentration of $2 \times 10^6$ cpm $ml^{-1}$, were hybridized to separate arrays according to the user manual. The membranes were exposed to Kodak X-ray films with intensifying screens at – 70°C for different time points. After hybridization, the results were analysed by autoradiography, normalizing the blots according to the transcriptional abundance of the housekeeping genes included on the array (ubiquitin, GAPDH, α-tubulin, β-actin, 23-kDa highly basic protein, ribosomal protein S9). As criteria for changes in gene expression, the intensity difference had to be at least twofold, whereas a more than fourfold difference was designated as strong alteration.

### Northern blot analyses

Total RNA from different passages of RUCH-2 cells was obtained as previously described under RT-PCR analyses. Five micrograms of total RNA were used per lane. The probes (inserts coding for cyclin A (EST clone 2394165), p21 (EST clone 470149), MMP-3 (EST clone 2113735) and the human TIMP-3, Myf3 and Myf5) were generated by random priming (Prime-a-gene, Promega) with $\alpha^{32}$P-dATP and used for hybridization at 68°C with QuickHyb Hybridization Solution (Stratagene) according to the manufacturer's instructions. The membrane was exposed to Kodak X-ray films and was reprobed thereafter.

### RESULTS

### Isolation of the cell line

To establish a cell line from the tumour specimen, mice were subcutaneously (s.c.) injected with minced tumour pieces. After 5 weeks, the tumour was resected and placed in culture. In parallel, some tumour material was injected again into a nude mouse. In culture, morphologically distinct cells grew rapidly out of the tumour pieces; however, the homogenous cell population finally resulting after several passages was identified as human fibroblasts by morphology, karyotype analysis, as well as RT-PCR experiments for myogenic markers (see below). Therefore this cell line was called FUCH-1 (for *F*ibroblast of *U*niversity *C*hildren's *H*ospital).

After the second xenograft reached 5 mm in diameter (3 months in total), it was minced and placed in culture. This time no human fibroblasts could be detected by microscopic examination. The cells appeared mostly elongated and spindle-shaped with a few multinucleated cells detectable. Molecular characterization clearly established that these cells are derived from the original tumour (see below). Compared to other RMS cell lines these cells are rather large. The resulting cell line was called RUCH-2 (*R*habdomyosarcoma of *U*niversity *C*hildren's *H*ospital) and is in continuous culture since 16 July 1997. As expected, the cells underwent a crisis in culture around passage 32–38 (~ 3.5 months in culture). They became extremely flat and almost stopped dividing. Then, they started to proliferate again which was accompanied by phenotypic changes to polygonal homogenous cells, resembling myogenic precursor cells (Figure 1). These morphological observations were later confirmed on the molecular level (see below).

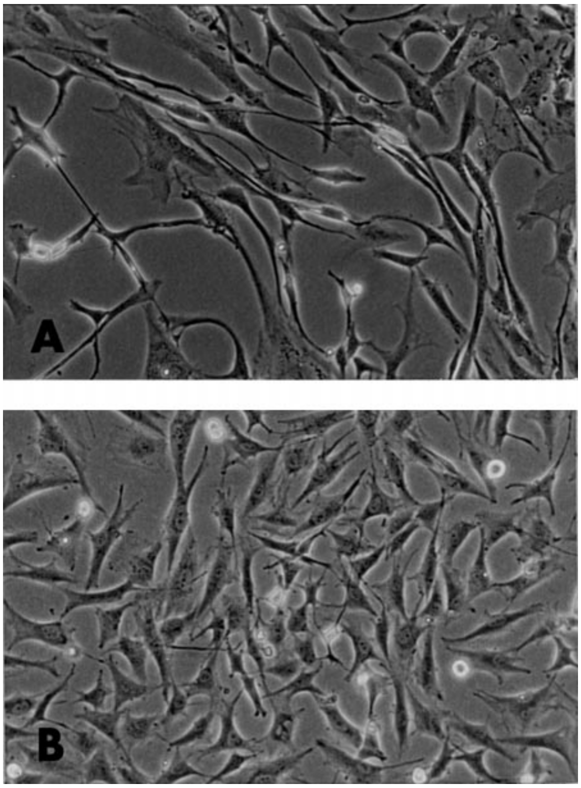

**Figure 1** Morphology of RUCH-2 cells. (**A**) Phase contrast picture of low passage (P22) and (**B**) high passage (P63) RUCH-2 cells (100 × magnification)

The RUCH-2 cells were split 1:2 every 65–72 h until passage 30 (P30), and every 48 h after P47. Interestingly, cells below P30 synthesized extensive extracellular matrix as evidenced by a gelatinous appearance of the culture supernatant, which disappeared after P38. These results demonstrate that we could successfully establish a new cell line derived from a botryoid rhabdomyosarcoma.

### Karyotype analyses of RUCH-2 cells

To better characterize and monitor possible changes in the RUCH-2 karyotype, cells were analysed at passage 3, 20, 32, 46, 62, 84 and 104 and compared with the original tumour. Cytogenetic investigation of the original tumour revealed a karyotype of:

54~57,X,–X,+7,+inv(7)(q11q32),+8,+8,+12,+13,+13,+19,+20,+21,+r,+0~2mar[cp6].

In comparison, initial analysis of the cell line (at P3) demonstrated a karyotype of:

55,X,–X,+7,+inv(7)(q11q32),+8,+8,+12,+13,+13,+19,+20,+21/55,idem,del(20)(q13)[cp40].

© 2000 Cancer Research Campaign 

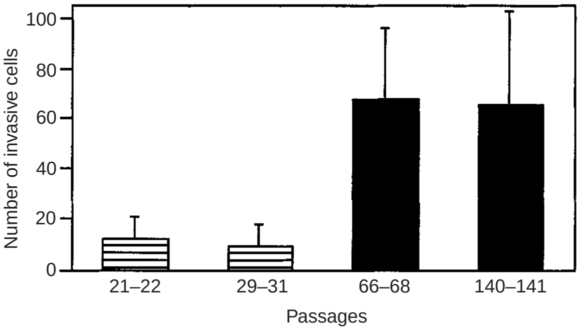

**Figure 2** In vitro invasiveness of RUCH-2 cells. Cells at different passages were analysed for their invasive behaviour. Low passage cells, P21–22 and P29–31 (striped bars), were compared with higher passages, P66–68 and P140–141 (black bars). The number of cells which invaded the matrigel was determined after staining with crystal violet and counting under the microscope. Data represent mean values ± s.e.m of three independent experiments

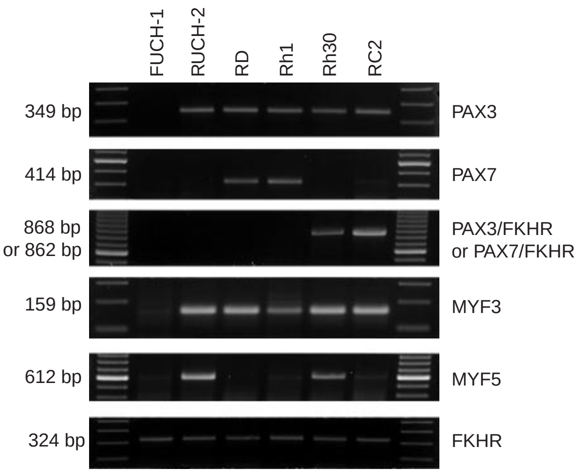

**Figure 3** Expression of Pax genes and members of the Myf gene family in human cell lines. One hundred nanograms of total RNA per RT-PCR reaction were used. All cell lines (RUCH-2, RD, Rh1, Rh30, and RC2) are RMS, except FUCH-1, which is a fibroblast line. FKHR was amplified as an internal control for RNA quality. RT-PCR products were electrophoresed in 1–2.5% agarose gels, stained with ethidium bromide. A 100-bp ladder was used as marker (strong band = 600 bp). The expected length of the PCR products are indicated at the left

Hence, this karyotype was essentially identical to the one of the original tumour. Further analysis at passages 20 and 32 displayed a similar karyotype without any major changes. Hyperdiploidy, as seen here, with few structural aberrations appears to constitute a distinct karyotype subgroup in RMS (Wang-Wuu et al, 1988). A different, earlier, study of ten eRMS tumour samples showed gain of the whole or a large part of various chromosomes, notably chromosomes 2 (60% of cases), 7 (50%), 8 (60%), 12 (60%), 13 (60%), 17 (40%), 18 (40%) and 19 (40%) (Weber-Hall et al, 1996). Hence, the original tumour and the cells up to P32 reflect many of these described alterations by displaying a gain of chromosomes 7, 8, 12, 13 and 19.

Interestingly, marked changes in the karyotype were observed starting from P46. Initially, we detected large cell to cell variation with the simplest clone being near triploid and containing multiple additional rearrangements. Also present was a distinct polyploid population and some metaphases with double minutes (dmin). Cells with dmin continued to be present at P62, but were no longer detected at P84. The final analysis at P104 showed a karyotype of:

67,X,add(X)(p11),–X,add(1)(q12)x2,del(1)(p34),+del(1)(p21), –2,t(3;12)(q21;p13),–6,–9,–9,add(9)(p12),–10,der(11)t(1;11) (p22;q22),add(12)(q13),der(12)t(3;12)(q21;p13),–13,–14,add (16)(q12),–17,add(18)(q23),der(18)t(?17;18)(q21;q23),+20, +20,–21,–22,– 22,+4~5mar[cp50].

The above karyotype showed at this point only limited cell to cell variation anymore. However, with the exception of the add(X)(p11) and the del(1)(q12), all major structural rearrangements were already present at P46. In contrast certain rearrangements identified at P46 were lost thereafter, such as a del(2)(p22), add(9)(q34) and add(17)(p11). These might have been apparent precursors for the subsequent loss of the entire chromosomes. Among the abnormalities acquired between P32 and P46 those of del(1p), t(3q), –9, add(9p) and add(18q23) have been previously reported and/or observed in primary RMS samples karyotyped at our hospital (data not shown). Hence, the RUCH-2 karyotype at late passage (P104) had karyotypic similarities to primary metastasis or relapse RMS tumours. Based on these results the changes of RUCH-2 cells observed during evolution in culture show some interesting parallels to normal RMS tumour progression.

### Induction of metastasis in late passage RUCH-2 cells

To examine their tumorigenic potential, $5 \times 10^6$ RUCH-2 cells at early and late passages were injected s.c. into nude mice. The control cell line FUCH-1, normal human fibroblasts (judged by karyotyping) of the same patient, did not lead to tumour growth even after 3.5 months of observation. In contrast RUCH-2 cells at P30 induced, in two out of three mice, tumours of 4–6 mm in diameter after 3.5 months, which is about a quarter of the size of tumours induced by the established RMS cell line RD. In none of the animals obvious metastases were observed.

Next, we used RUCH-2 cells at P67. Whereas tumours grew to the same size as cells at P30 in all three mice, one animal had to be sacrificed after ~2.5 months because of metastasis. After dissection numerous huge metastases were observed in the liver and the spine. Both, the xenographed tumours and the liver metastases were confirmed to be of RMS origin by immunohistochemical investigations demonstrating desmin, myoglobin and α-skeletal actin staining (data not shown), whereby the metastasis showed more intense staining. A similar result was obtained with a second animal, whereas only a small tumour grew in a third animal. In contrast, no metastases were detected in mice injected with RD cells. These results suggest that RUCH-2 cells progressed in culture to acquire a metastatic phenotype.

### In vitro invasivity of the RUCH-2 cell line

The notion that metastases were observed in two out of three mice injected with late passage RUCH-2 cells suggested that the cells gained metastatic properties including the ability to invade extracellular matrix. To confirm this suggestion in vitro, modified Boyden chambers were used to measure the ability of the cells to invade extracellular matrix. At P21–31, 8.7 to 11.5 cells invaded the matrigel, in contrast to P66–141 where 65.5 to 67.7 cells were counted (see Materials and Methods) (Figure 2). Hence, late passage cells were significantly (eightfold) more invasive than cells at early passages supporting the observation that they acquired a more invasive and metastatic phenotype.

 © 2000 Cancer Research Campaign

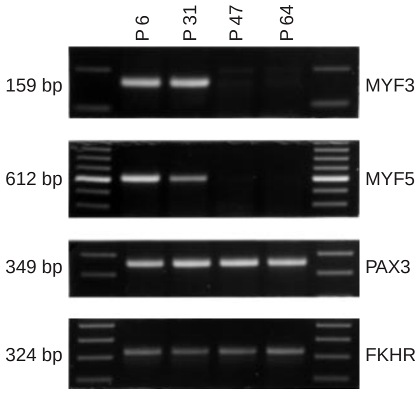

**Figure 4** Expression of myogenic regulators at different passages of RUCH-2 cells. RT-PCR reactions were performed with RNA isolated from cells at different passages. FKHR was amplified as an internal control for RNA quality. RT-PCR products were loaded on agarose gels and stained with ethidium bromide. As marker a 100-bp ladder was used (strong band = 600 bp). The expected length of the PCR products are indicated at the left

### Molecular characterization of RUCH-2

To get insight into possible molecular changes accompanying the observed biological behaviour of RUCH-2 cells, we first analysed the expression pattern of regulatory proteins known to be involved in muscle differentiation. RT-PCR analyses were performed to characterize possible changes in the expression of PAX transcriptional regulators and myogenic determination factors (Myf). As controls, cell lines were used which provide either positive or negative controls for every RT-PCR reaction. FKHR was amplified as a control for RNA quality. As shown in Figure 3, similar FKHR levels could be detected in all samples, indicating integrity of the RNAs. RT-PCR results further showed that RUCH-2 cells express PAX3, similar to other RMS cell lines (RD, Rh1, Rh30 and RC2). Only the corresponding fibroblast cell line did not express PAX3 confirming the aberrant expression of PAX3 in RMS. Neither translocation products (PAX3/FKHR, PAX7/FKHR) nor PAX7 expression could be detected in RUCH-2 cells. Myf3 expression is assumed to be characteristic for RMS tumours and cell lines (Clark et al, 1991) and was also detected in RUCH-2 (Figure 3, lane 2). In addition, Myf5 is expressed in RD and RUCH-2 cells, whereas Myf4 was detected in RD, RC2, and very weakly in Rh30 but not in RUCH-2 and Rh1 cells (data not shown). Since both myogenic markers Myf3 and Myf5 were expressed in RUCH-2 cells at P3, the myogenic origin of the RUCH-2 cell line could be confirmed on the molecular level.

To monitor possible progression in culture, RT-PCR investigations were repeated at passage 6, 31, 47, and 64 (Figure 4). Interestingly, Myf3 and Myf5 transcripts were no longer detected after P31 (results confirmed by Northern blot analyses, see Figure 5), whereas FKHR and PAX3 expression remained constant. Hence, the appearance of a metastatic population was associated with changes in the karyotype as well as alterations in the expression levels of myogenic regulatory factors, Myf3 and Myf5, whose expression was down-regulated in late passage RUCH-2 cells.

### Differential gene expression in early and late passage RUCH-2 cells

To monitor global changes in expression pattern of genes likely to be involved in tumour progression, expression of 588 cancer and metastasis-related genes were simultaneously examined using cDNA microarray hybridization. Gene expression was monitored using $^{32}$P-labelled cDNA generated from RNA isolated from cells at P11 and P63. Ninety-two genes displayed altered expression levels between non-metastatic and metastatic cells, representing almost every sixth gene investigated. Of these 92 genes, 64 were down-regulated in high passage RUCH-2 cells, 17 of them strongly. In contrast, 28 investigated cDNAs were up-regulated including five strong ones. To facilitate the identification of potentially important genes and because of the large number of alterations observed, three functional categories were selected and examined in more detail, namely apoptosis-related genes, proteases and protease inhibitors, and cell cycle regulators. Genes from these categories showing altered expression are listed in Table 1. The complete list of the 92 differentially expressed genes in all categories can be viewed at our homepage: *http://www.unizh.ch/kispi/clinchem_html/schafer.html*. To independently confirm the alterations observed in the microarray, additional Northern blot analyses were performed using several different passages of RUCH-2 RNA (Figure 5). These experiments revealed that the expression of genes analysed from different functional groups strictly correlated with expression data from the microarray study: up-regulation of MMP-3 and, to a lesser extent, cyclin A, down-regulation of TIMP-3 and p21. Additionally, loss of expression of myogenic markers, as seen in RT-PCR experiments (Figure 3), could be confirmed quantitatively (Myf3, Myf5).

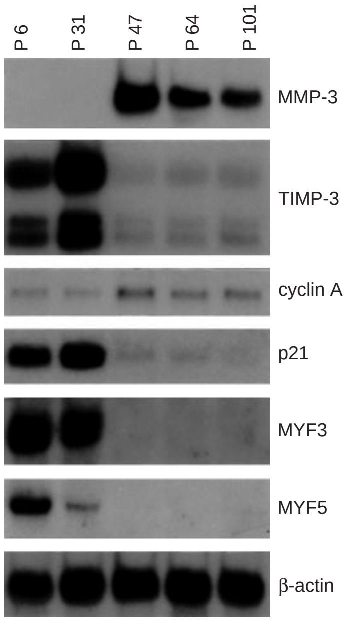

**Figure 5** Northern blot analyses of RUCH-2 cells at different passages. The indicated genes (MMP-3, TIMP-3, cyclin A, p21, MYF3, MYF5) were analysed sequentially on the same blot. TIMP-3 showed a downregulation for all three splice variants. The amount of total RNA was confirmed with a β-actin probe

Looking at the differentially expressed genes in Table 1, extensive changes in the composition of the extracellular matrix were observed. Furthermore, as could probably be expected, this was accompanied by down-regulation of tissue inhibitors of metalloproteinases (TIMP) and up-regulation of some matrix metalloproteinases (MMP). Genes of particular interest might include MMP-14 and TIMP-2, since coexpression of both genes during

© 2000 Cancer Research Campaign

**Table 1** Differential gene expression (RUCH-2 P63 compared to RUCH-2 P11)

| | Gene | |
|---|---|---|
| **Functional category[a]** | **Down-regulated** | **Up-regulated** |
| Apoptosis-related | caspase-2 | caspase-4 + caspase-5 |
| | TNFR1 | Akt-1 |
| | A1[c] | |
| | Bag-1[c] | |
| | DR3[c] | |
| | CD27[c] | |
| Proteases and inhibitors | MMP-14 | MMP-3[c] |
| | TIMP-1 | MMP-8[c] |
| | TIMP-2 | PAI-2[c] |
| | TIMP-3 | |
| | EMMPRIN | |
| | t-PA | |
| | LRP | |
| Cell cycle regulators[b] | cyclin D1 | Cyclin A |
| | cyclin G1 | Cyclin B1 |
| | p21[c] | |

[a]Only selected categories and [b]genes, entire list presented on our homepage:*http://www.unizh.ch/kispi/clinchem_html/schafer.html*; [c]strong alterations.

muscle development has been reported (Apte et al, 1997). In addition, the extracellular matrix metalloproteinase inducer (EMMPRIN), expressed on the cell surface, was down-regulated. Its absence in the vertical growth phase and in metastatic lesions of melanomas suggests that other factors are involved in tissue degradation during later stages of tumour progression in malignant melanoma (van den Oord et al, 1997). Furthermore, strong up-regulation of plasminogen activator inhibitor 2 (PAI-2) was coordinated with a decreased expression of tissue-type plasminogen activator (t-PA). Indeed low t-PA and high PAI-2 levels have been correlated with poor overall survival in a number of primary tumours of different origin (Andreasen et al, 1997).

Interestingly, we also observed strong down-regulation of death receptors belonging to the tumour necrosis factor and nerve growth factor superfamily, namely DR3, CD27 and TNFR1. This suggests a possible hampered response of high passage RUCH-2 cells to appropriate apoptotic stimuli. In support of this, the signalling kinase Akt-1, a strong mediator of cell survival, is overexpressed as well in late stage RUCH-2 cells. However, contrasting this potential progression towards apoptosis resistance, two anti-apoptotic molecules, bag-1 and A1 (bfl-1) were also down-regulated. Hence, not single alterations but a complex coordinated up- and down-regulation of molecules involved in regulation of apoptosis was observed, which might shift the balance of the cellular response into one particular direction. This can also be seen when analysing genes involved in cell cycle regulation. Most prominently, cyclin A and cyclin B1 were up-regulated (stimulating progression through the cell cycle), whereas cyclin D1 as well as cyclin G1 were down-regulated (slowing progression). Furthermore, a striking down-regulation is seen for the cell cycle inhibitor p21 (again stimulating progression). Here, as a net result from these complex alterations, the proliferation rate of the cell population increases.

In summary, we identified a large number of genes whose expression is altered during RUCH-2 progression. Some of these might play a predominant role in tumour progression towards the metastatic phenotype that can now be further evaluated in additional investigations, involving primary tumour samples.

### DISCUSSION

In this work we describe the isolation and molecular characterization of a new human cell line derived from a botryoid RMS. The key feature of this new cell line is its availability at low, non-metastatic and high, metastatic, passages following a spontaneous progression in vitro. This provides an interesting cellular model, since cells could initially be established from the original tumour not under treatment at time of biopsy.

Unlike other cell culture models of metastasis which are mostly based on single clone selection, progression observed in the RUCH-2 cell line occurred spontaneously in the population. We provide here three different experimental criteria demonstrating this evolution: changes in morphology and enhanced growth rate, gain of invasiveness through matrigel in vitro and, finally, de novo appearance of metastases in a nude mouse model in vivo.

How does tumour progression in culture relate to in vivo progression? Genome instability and resulting selection of subpopulation underlie progression in vitro as well as in vivo. However, because of culture conditions, different subpopulations might be selected. To assess this notion frequent examinations of the tumour cell karyotype were carried out. These cytogenetic investigations revealed that the karyotype in early passages was very similar to the original tumour. However, in later passages, a number of chromosomal gains and rearrangements were observed. Strikingly, several of the breakpoints which were newly observed, namely 1p21, 3q21, 12p13, 12q13 and 17q21 seem to be non-random, since they were also reported in primary RMS tumour samples (Whang-Peng et al, 1992; Kullendorff et al, 1998). This also includes a rather rare breakpoint at 18q23. Although comparison between in vitro and in vivo progression is clearly very limited, RUCH-2 cells represent, to our knowledge, the only progression model for human RMS available and hence provide a starting point for the identification of novel genes involved in rhabdomyosarcoma progression.

To begin to investigate possible molecular alterations in progression, we first performed RT-PCR experiments to study the expression levels of selected known regulatory molecules. While RUCH-2 cells at all passages expressed PAX3, the expression of the myogenic regulators Myf3 and Myf5 was restricted to early passages and completely disappeared later on as demonstrated by RT-PCR and quantitative Northern blot analysis. These results have implications for other tumour cell lines which are thought to be of RMS origin, since doubts have been expressed over their origin based on the missing expression of the myogenic regulatory proteins. Examples of such publicly available cell lines are A204 and Hs729. Our results suggest that they should not be excluded as having RMS origin, especially when they have been cultivated for an extensive period. On the other hand, analysis of Myf genes in primary RMS tumours revealed that only 18 out of 20 samples displayed Myf gene expression (Clark et al, 1991). Interestingly, a second RMS cell line established in our group, RUCH-3, which was derived from a relapse embryonal RMS, did never express Myf3 and Myf5, but PAX3 (the patient died a short time after excision). Hence, lack of Myf3 might be confined to more advanced tumour stages.

 *© 2000 Cancer Research Campaign*

To provide a more comprehensive analysis of the molecular changes involved in metastatic progression, we then applied the cDNA microarray technique to compare expression of 588 known tumour-associated genes from late with early passage cells. The microarray hybridization most likely reflects true changes in expression level, because the alteration of selected genes like TIMP-3, MMP-3, cyclin A and $p21^{WAF1}$ could be confirmed with quantitative Northern blot analyses of RUCH-2 cells at various passages (Figure 5). As expected, a number of alterations (either up- or down-regulation) were observed in the composition of the extracellular matrix, including enhanced expression of proteinases. However, other alterations might provide novel insight into RMS development. Some of the most striking changes were observed in the category of apoptosis-related genes, where the down-regulation of three receptors of the TNF receptor family, namely TNFR1, DR3 and CD27, was very prominent. So far, neither the expression nor the role of these death receptors has been investigated in skeletal muscle cells. Down-regulation of death receptors has, however, been correlated with the development of other cancers, e.g. TNFR1 in non-small-cell lung cancer (Wyllie, 1997; Tran et al, 1998). Furthermore, an enhanced resistance of metastatic cancer cells to programmed cell death has been described for several neoplasms (Glinsky et al, 1997; Takaoka et al, 1997). Hence, these receptors could play an important role in RMS development as well. Interestingly, TNFR1 and CD27 are both located at 12p13, a locus where rearrangements have been observed in high passage RUCH-2 cells, raising the possibility that their expression might be influenced by the translocation event.

A more extensive use of DNA microarray technology should undoubtedly allow the identification of additional changes important for RMS progression in the near future. Evidently, all changes will have to be verified in additional RMS cells and, ideally, biopsy samples from primary tumours as well as metastases from the same patient. Nevertheless, the RUCH-2 cell line provides a starting point for the identification of progression associated genes in RMS, genes which might be important for other tumours as well.

### ACKNOWLEDGEMENTS

We thank Prof. CW Heizmann and Prof. FE Würgler for the continuous support, Dr A Feldges (Children's Hospital, St. Gallen, Switzerland) for providing us with the primary tumour material and transmitting the follow-up data. The characterization of the metastases in mice has been kindly performed by Dr T Stallmach (Pathology, University Hospital of Zurich). This work was supported by the Krebsforschung Schweiz and Swiss National Science Foundation (31-46886.96 and 31-56869.99).

### REFERENCES

Anand G, Shapiro DN, Dickman PS and Prochownik EV (1994) Rhabdomyosarcomas do not contain mutations in the DNA binding domains of myogenic transcription factors. *J Clin Invest* **93**: 5–9

Andreasen PA, Kjoller L, Christensen L and Duffy MJ (1997) The urokinase-type plasminogen activator system in cancer metastasis: a review. *Int J Cancer* **72**: 1–22

Apte SS, Fukai N, Beier DR and Olsen BR (1997) The matrix metalloproteinase-14 (MMP-14) gene is structurally distinct from other MMP genes and is co-expressed with the TIMP-2 gene during mouse embryogenesis. *J Biol Chem* **272**: 25511–25517

Barr FG, Galili N, Holick J, Biegel JA, Rovera G and Emanuel BS (1993) Rearrangement of the PAX3 paired box gene in the paediatric solid tumour alveolar rhabdomyosarcoma. *Nat Genet* **3**: 113–117

Barr FG, Nauta LE and Hollows JC (1998) Structural analysis of Pax3 genomic rearrangements in alveolar rhabdomyosarcoma. *Cancer Genet Cytogenet* **102**: 32–39

Betts DR, Koesters R, Plüss H-J and Niggli FK (1997) Routine karyotyping in Wilms' tumor. *Cancer Genet Cytogenet* **96**: 151–156

Clark J, Rocques PJ, Braun T, Bober E, Arnold HH, Fisher C, Fletcher C, Brown K, Gusterson BA, Carter L and Cooper CS (1991) Expression of members of the myf gene family in human rhabdomyosarcoma. *Br J Cancer* **64**: 1039–1042

Davis RJ, D'Cruz CM, Lovell MA, Biegel JA and Barr FG (1994) Fusion of PAX7 to FKHR by the variant t(1;13)(p36;q14) translocation in alveolar rhabdomyosarcoma. *Cancer Res* **54**: 2869–2872

Galili N, Davis RJ, Fredericks WJ, Mukhopadhyay S, Rauscher III FJ, Emanuel BS, Rovera G and Barr FG (1993) Fusion of a fork head domain gene to PAX3 in the solid tumour alveolar rhabdomyosarcoma. *Nat Genet* **5**: 230–235

Glinsky GV, Glinsky VV, Ivanova AB and Hueser CJ (1997) Apoptosis and metastasis: increased apoptosis resistance of metastatic cancer cells is associated with the profound deficiency of apoptosis execution mechanisms. *Cancer Lett* **115**: 185–193

ISCN (1995): *An International System for Human Cytogenetic Nomenclature*. Mitelman F (ed). S Karger: Basel

Kullendorff CM, Donner M, Mertens F and Mandahl N (1998) Chromosomal aberrations in a consecutive series of childhood rhabdomyosarcoma. *Med Pediatr Oncol* **30**: 156–159

Ludolph DC and Konieczny SF (1995) Transcription factor families: muscling in on the myogenic program. *FASEB J* **9**: 1595–1604

McAllister RM, Melnyk J, Finkelstein JZ, Adams EC and Gardner MB (1969) Cultivation in vitro of cells derived from a human rhabdomyosarcoma. *Cancer* **24**: 520–526

Nanni P, Schiaffino S, De Giovanni C, Nicoletti G, Prodi G, Del Re B, Eusebi V, Ceccarelli C, Saggin L and Lollini P-L (1986) RMZ: a new cell line from a human alveolar rhabdomyosarcoma. In vitro expression of embryonic myosin. *Br J Cancer* **54**: 1009–1014

Newton WA, Gehan EA, Webber BL, Marsden HB, van Unnik AJM, Hamoudi AB, Tsokos MG, Shimada H, Harms D, Schmidt D, Ninfo V, Cavazzana AO, Gonzalez-Crussi F, Parham DM, Reiman HM, Asmar L, Beltangady MS, Sachs NE, Triche TJ and Maurer HM (1995) Classification of rhabdomyosarcomas and related sarcomas. *Cancer* **76**: 1073–1085

Pappo AS (1995) Rhabdomyosarcoma and other soft tissue sarcomas of childhood. *Curr Opin Oncol* **7**: 361–366

Schäfer BW, Czerny T, Bernasconi M, Genini M and Busslinger M (1994) Molecular cloning and characterization of a human PAX-7 cDNA expressed in normal and neoplastic myocytes. *Nucleic Acids Res* **22**: 4574–4582

Scrable HJ, Witte DP, Lampkin BC and Cavenee WK (1987) Chromosomal localization of the human rhabdomyosarcoma locus by mitotic recombination mapping. *Nature* **329**: 645–647

Shapiro DN, Sublett JE, Li B, Downing JR and Naeve CW (1993) Fusion of PAX3 to a member of the forkhead family of transcription factors in human alveolar rhabdomyosarcoma. *Cancer Res* **53**: 5108–5112

Takaoka A, Adachi M, Okuda H, Sato S, Yawata A, Hinoda Y, Takayama S, Reed JC and Imai K (1997) Anti-cell death activity promotes pulmonary metastasis of melanoma cells. *Oncogene* **14**: 2971–2977

Tonin PN, Scrable H, Shimada H and Cavenee WK (1991) Muscle-specific gene expression in rhabdomyosarcomas and stages of human fetal skeletal muscle development. *Cancer Res* **51**: 5100–5106

Tran TA, Kallakury BV, Ambros RA and Ross JS (1998) Prognostic significance of tumor necrosis factors and their receptors in nonsmall cell lung carcinoma. *Cancer* **83**: 276–282

van den Oord JJ, Paemen L, Opdenakker G and de Wolf-Peeters C (1997) Expression of gelatinase B and the extracellular matrix metalloproteinase inducer EMMPRIN in benign and malignant pigment cell lesions of the skin. *Am J Pathol* **151**: 665–670

Wang-Wuu S, Soukup S, Ballard E, Gotwals B and Lampkin B (1988) Chromosomal analysis of sixteen human rhabdomyosarcomas. *Cancer Res* **48**: 983–987

Weber-Hall S, Anderson J, McManus A, Abe S, Nojima T, Pinkerton R, Pritchard-Jones K and Shipley J (1996) Gains, losses and amplification of genomic material in rhabdomyosarcoma analyzed by comparative genomic hybridization. *Cancer Res* **56**: 3220–3224

Whang-Peng J, Knutsen T, Theil K, Horowitz ME and Triche T (1992) Cytogenetic studies in subgroups of rhabdomyosarcoma. *Genes Chromosomes Cancer* **5**: 299–310

Wyllie AH (1997) Apoptosis and carcinogenesis. *Eur J Cell Biol* **73**: 189–197

© 2000 Cancer Research Campaign 
